# Monocular lens dislocation due to vomiting-a case report

**DOI:** 10.1186/s12886-017-0651-8

**Published:** 2018-01-08

**Authors:** Min Wang, Yufang Gao, Rong Li, Sheng Wang

**Affiliations:** 1 Department of Ophthalmology, the First People’S Hospital of Xian yang, #10 Biyuan Road, Xian yang, Shaanxi 712000 China; 2 Department of Laboratory Diagnosis, the Central Hospital of Xian yang, #78 Renmin East Road, Xian yang, Shaanxi 712000 China; 3 Department of Ophthalmology, The First Affiliated Hospital, Xi’an Medical University, #48 Fengao West Road, Xi’an, Shaanxi 710000 China

**Keywords:** Lens dislocation, Vomitting, Case report

## Abstract

**Background:**

Lens dislocation is a common disease in ophthalmology, which leads to vision loss, while the lens dislocation caused by vomiting has not been reported yet. We report a case of lens dislocation caused by simple vomiting. This case further implicated for the pathogenesis of lens dislocation.

**Case presentation:**

A 51-year-old male who complained about “dizziness, vomiting, and the vision decreased for 4 h in right eye”, after the eye examination, he was been diagnosed with “lens dislocation induced by simply vomiting “. Surgery was performed successfully.We highlight the pathogenesis and development of the lens dislocation in this rare condition.

**Conclusion:**

Lens dislocation could be induced by simple vomitting, which increased the vitrous cavity presure to shock the zonular fiber and push the lens into the anterior chamber.

## Background

Lens dislocation could be caused by many reasons and the most common one is ocular trauma [1], followed by ocular surgery and lens spontaneous dislocation due to hypermature cataract [2]. Lens dislocation could also occurred in some congenital dysplasias, such as Marfan syndrome, Marchesani syndrome, and homocystinuria [3–9]. In addition, spontaneous lens dislocation has been frequently reported, but mostly in patients with chronic uveitis or high myopia [10, 11]. Ocular trauma and surgery exert an external force on the lens zonular fiber, to make it rupture and cause lens dislocation or subluxation. The vast majority of the eyes of patients with congenital dysplasia have binocular lens and zonular dysplasia, usually accompanied by systemic dysplasia at the same time [6–8], but the monocular lens dysplasia with systemic dysplasia is rarely reported. Therefore, lens dislocation or subluxation in such patients tends to be caused by the direct action of external force on the eyes.

Lens dislocation in those conditions mentioned above is very common in clinical practice. However, a simple vomiting directly leading to the monocular lens dislocation into the anterior chamber has not been reported yet. Thus, we presented such a case in this paper as the following.

## Case presentation

A male patient aged 51 years was admitted to our hospital on March 15, 2016 with a complaint of blurred vision in the right eye for 4 h after dizziness and vomiting. He felt dizzy when he got up and then symptoms of nausea and vomiting appeared. After he sit back and rested immediately, he felt decreased vision in his right eye. He has no history of trauma and eye diseases. His vision acuity was 20/20 in both eyes after the physical examination just 1 month ago. He was healthy with no history of systemic diseases, such as high blood pressure, diabetes, and heart disease. Normal development of his body, without signs of Marfan syndrome, Marchesani syndrome, and homocystinuria.

**Fig. 1:**
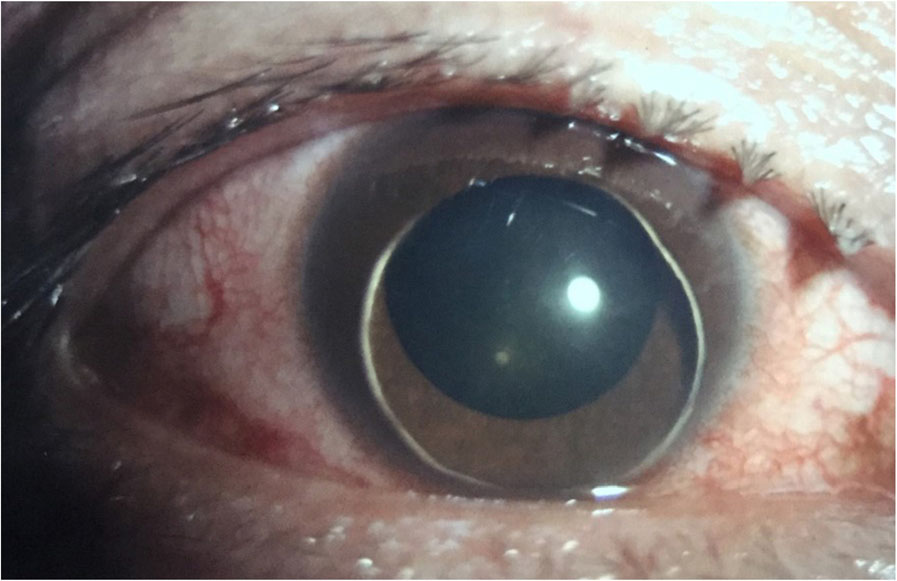
Lens dislocation into the anterior chamber in the right eye and obvieous conjunctival congestion

**Fig. 2:**
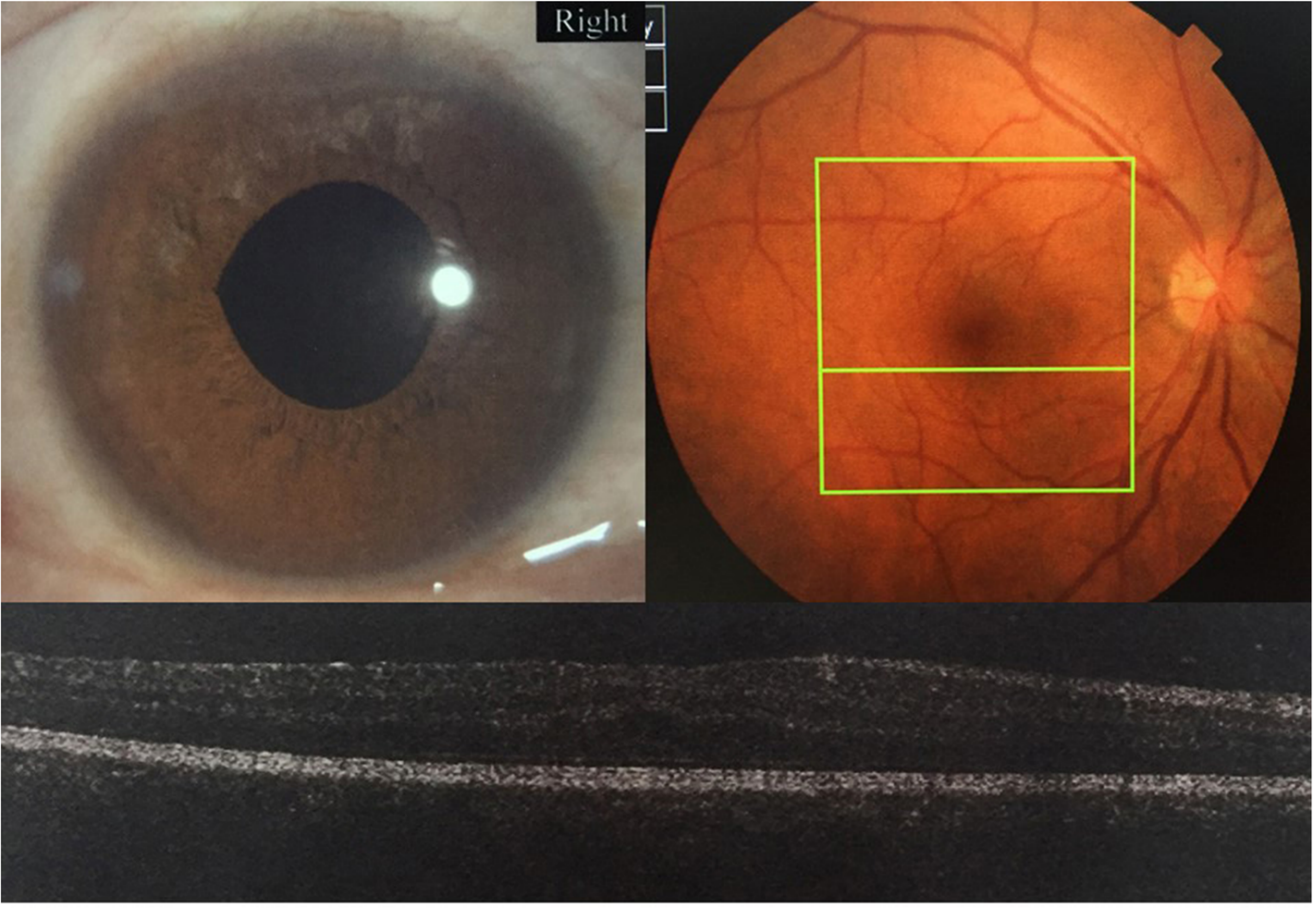
The pupil diameter returned to normal, the retina turned out to be normal and slight macular edema due to aphakia at 1 month after surgery

**Fig. 3:**
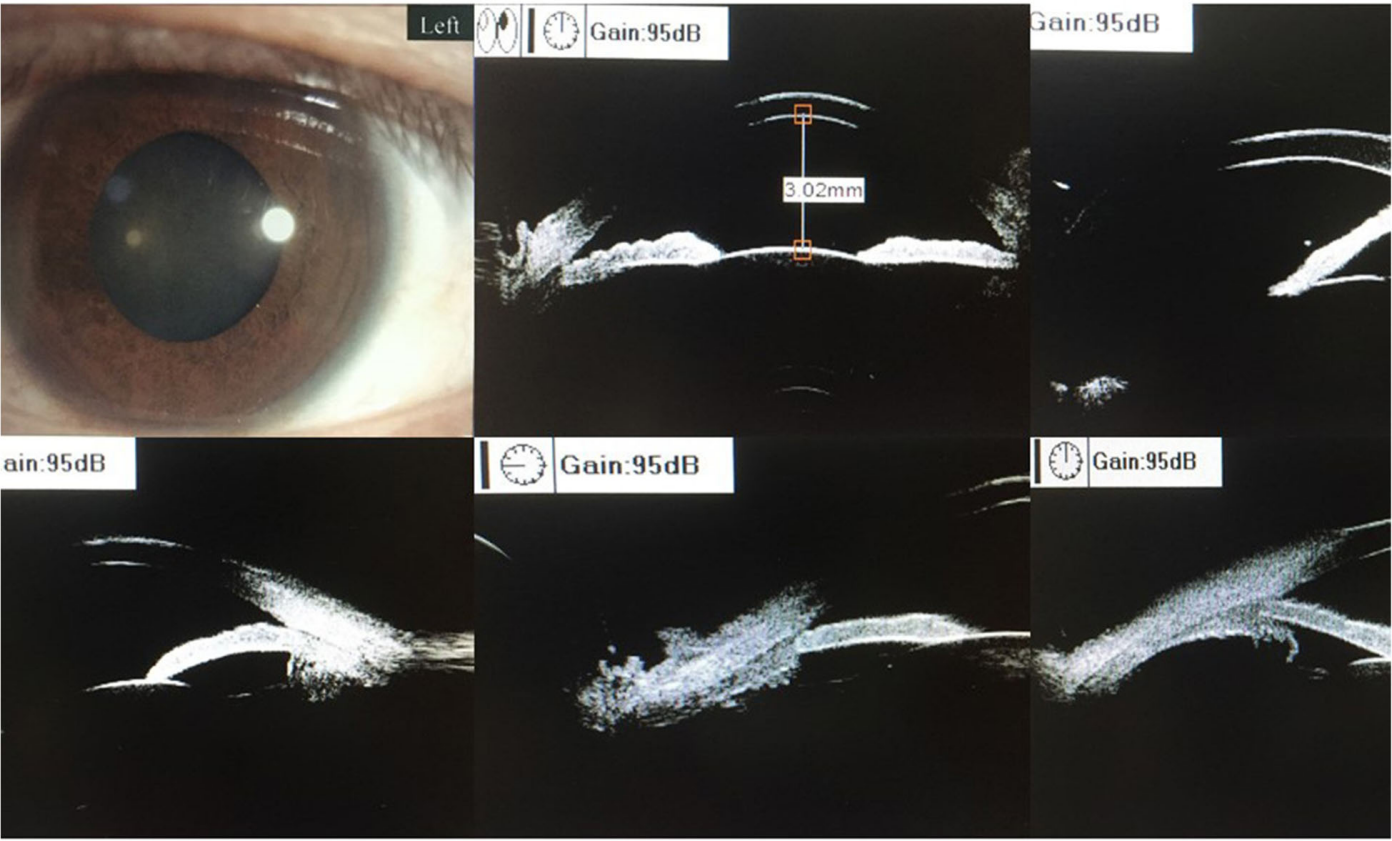
Normal eyeball development of the fellow eye (left), not spherophakia and normal ciliary body-suspensory ligament at all the four quadrants on ultrabiomicroscopy (UBM) check (sagittal view, 3:00, 6:00, 9:00 and 12:00 clock images in sequence,respectively)

**Fig. 4:**
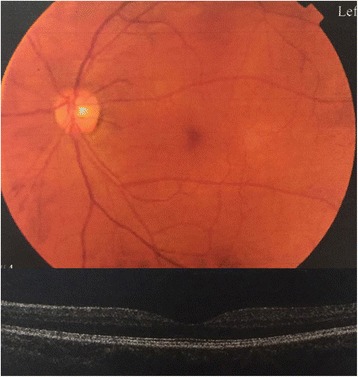
Normal funds imaging and optical coherence tomography (OCT) imaging of the left eye

**Fig. 5:**
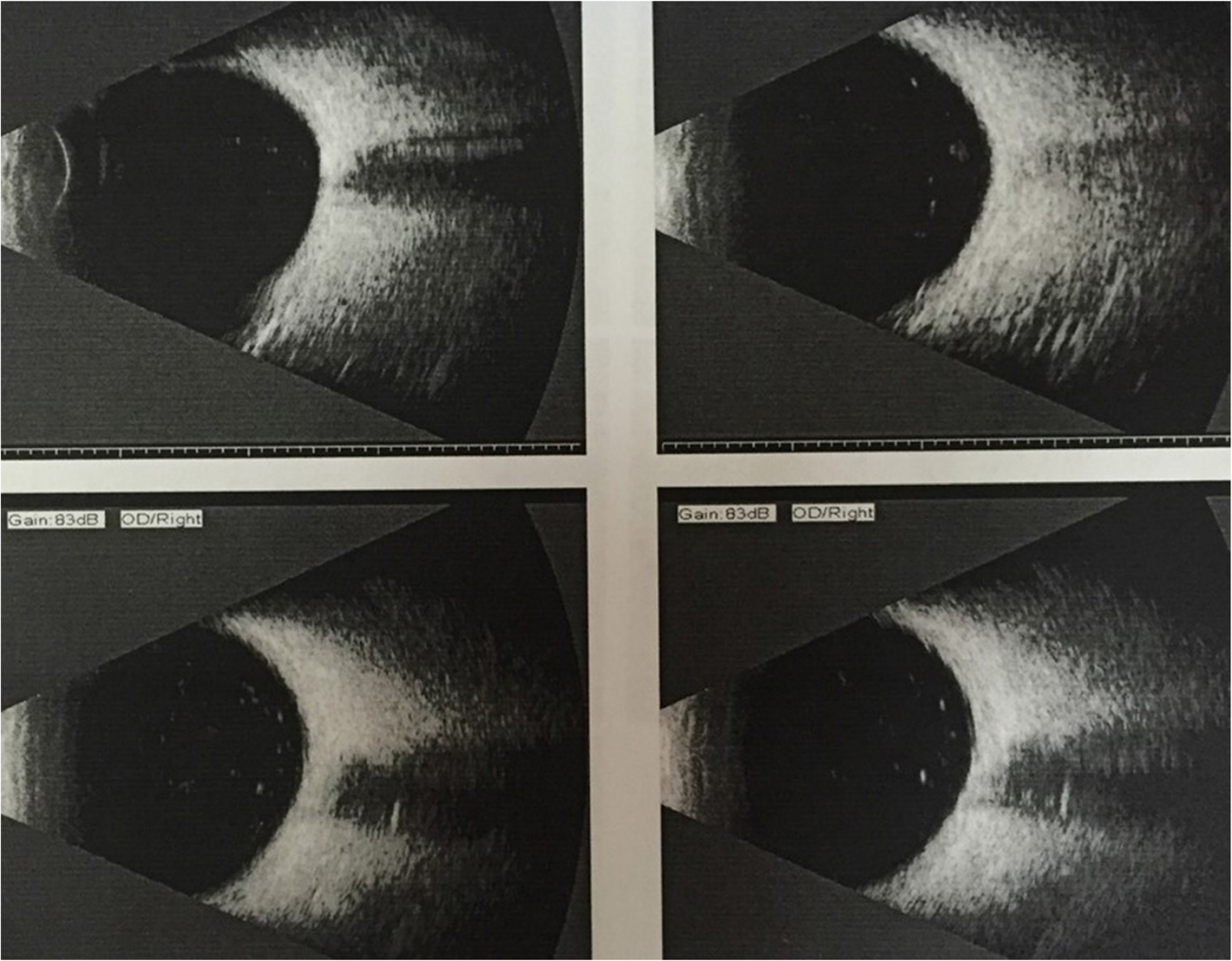
Normal B ultrasound of the left eye

**Fig. 6:**
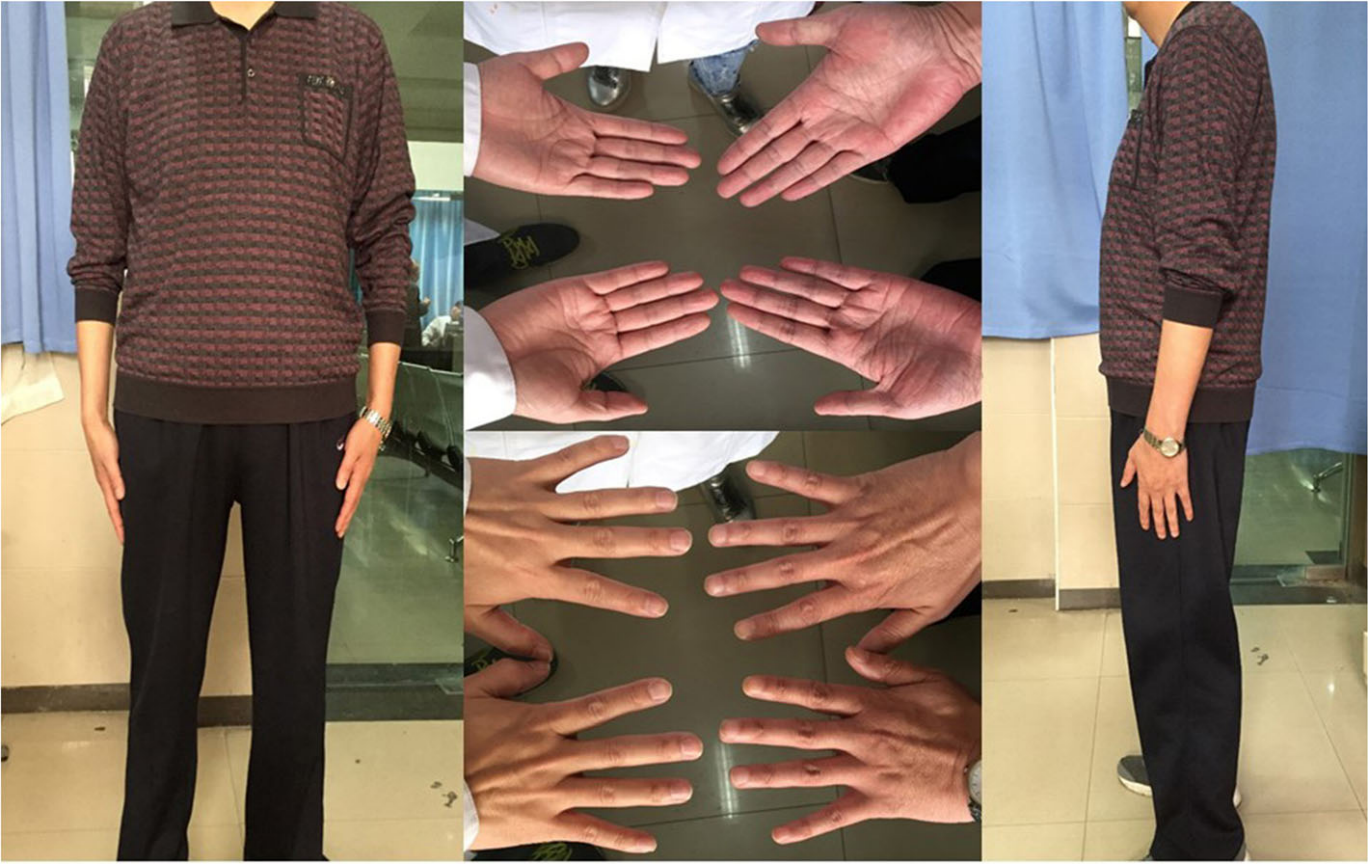
Normally developed hands compared with normal people (left: hands of the doctor, right: hands of the patient) and normal somatotype of the patient

No abnormality was found by general physical examination. His vision acuity was hand moving (HM) in right eye and 20/25 in left eye. The intraocular pressure in the right eye and the left eye was 20 mmHg (1 mmHg = 0.133 kPa) and 18 mmHg, respectively. Conjunctival congestion was noticed in right eye, however, the cornea is clear. The lens dislocated into the anterior chamber. The pupil was 6 mm in diameter and the light reflex was delayed. A small amount of vitreous body was seen behind the pupil (Fig. 1) and the retina was not clear. The left eye was normal. The patient was diagnosed as lens dislocation in right eye and perforned the surgery of lens removal combined with anterior vitrectomy after 48 h. The operation was succeed, but the corneal edema was obvious after surgery since the lens contacted with the corneal endothelia for long time. After treatment for 1 week, the cornea recoverd transparent and the anterior chamber was clear. The pupil diameter returned to normal after 1 month and the retina turned out to be normal (Fig. 2). The contralateral eye was normal when checked and no abnormality of the lens and the zonular fiber was found (Figs. 3, 4 and 5). His physical examination included the cardiac color ultrasound, the aortic doppler, and the brain magnetic resonance examination, was totally normal. His hands and somatotype were also normal (Fig. 6). His right vision was still HM (no improvement when corrected) and the IOP was 16 mmHg on the day of hospital discharge (one week after surgery). After 1 month, his corrected vision acuity was improved to 20/25 and the secondary intraocular lens suture fixation was performed 3 months later.

## Discussion

Vomiting is a violent process by which the stomach capacity is discharged through the mouth, and its characteristic is feeling sick, and then a series of coordinated abdominal muscle contraction and reverse esophageal peristalsis occur. Vomiting is caused by many reasons and the most common mechanism is that the tongue, throat, gastrointestinal mucosa, peritoneum, uterus,and inner ear balance organ are stimulated and neural impulse is transmitted to the medulla vomiting center [12]. Vomiting due to dizziness is mainly caused by the stimulation of the inner ear balance organ. Its clinical manifestation is special abdominal discomfort, accompanied by vagus nerve excitement symptoms, such as dizziness, salivate, slow pulse, and low blood pressure [13]. As Pasquale LR. et al. [14] reported, increase of the neck and chest pressure can result in increased intraocular pressure. The mechanism for this was that increase of neck and chest pressure hindered the jugular vein blood flow, therefore the blood in the head could not return to the heart. Currently, no related researches can be retrieved about the changes of intracranial pressure, orbital pressure, and intraocular pressure during vomiting. Based on the above theories, chest pressure was increased due to contractions of diaphragmatic muscle and abdominal muscle when vomiting, which could cause blocked venous reflux in the head, and eventually lead to transient increase of the orbital pressure, intraocular pressure, and especially the vitreous cavity pressure from back to forward.

In this case, the patient had no history of ocular trauma and his intelligence was normal. No abnormality was found in his skeletal system, cardiovascular system and urinary system. In his family, no similar patient has been found. In view of these conditions and his normal contralateral eye, developmental or congenital diseases were not considered. Therefore, since the patient has no history of trauma and surgery, we concluded that lens dislocation in his right eye was caused by vomiting due to dizziness which may be on account of orthostatic hypotension.

## Conclusion

The possible mechanism was that abdominal muscles and diaphragmatic muscles contracted instantly when vomiting occurred to bring about increased pressure in the head, orbit, and vitreous cavity to shock the zonular fiber and then make them rupture completely and subsequently the lens fell into the anterior chamber.

## Abbreviations

HM: Hand movement
IOP: Intra-ocular pressure
OCT: Optical coherence tomography
UBM: Ultrasound biomicroscopy
